# A comprehensive evaluation of single nucleotide polymorphisms associated with gastric cancer risk: A protocol for systematic review and network meta-analysis

**DOI:** 10.1097/MD.0000000000020448

**Published:** 2020-06-19

**Authors:** Zhuo-Miao Ye, Qing-Yu Hu, Jing-Hui Zheng, Chi Zhang, Xiang-Dong Zhu, You-Ming Tang

**Affiliations:** a Ruikang School of Clinical Medicine, Guangxi University of Chinese Medicine; b Department of Cardiology, Ruikang Hospital Affiliated to Guangxi University of Chinese Medicine; c Graduate School, Guangxi University of Chinese Medicine; d Department of Oncology; e Department of Gastroenterology, Ruikang Hospital Affiliated to Guangxi University of Chinese Medicine, Nanning, Guangxi, China.

**Keywords:** case-control study, gastric cancer, model of inheritance, network meta-analysis, susceptibility

## Abstract

**Background:**

Single nucleotide polymorphisms (SNPs) have been inconsistently associated with gastric cancer (GC) risk. This meta-analysis aimed to synthesize relevant data on SNPs associated with GC.

**Methods:**

Databases were searched to identify association studies of SNPs and GC published through January 2020 from the databases of PubMed, Web of Science, Embase, Cochrane Library, China National Knowledge Infrastructure, the Chinese Science and Technology Periodical Database, and Wan fang databases. Network meta-analysis and Thakkinstian algorithm were used to select the most appropriate genetic model, along with false positive report probability for noteworthy associations. The methodological quality of data was assessed based on the STrengthening the REporting of Genetic Association Studies statement Stata 14.0 will be used for systematic review and meta-analysis.

**Results:**

This study will provide a high-quality evidence to find the SNP most associated with GC susceptibility and the best genetic model.

**Conclusions:**

This study will explore which SNP is most associated with GC susceptibility.

**Registration:**

INPLASY202040132.

## Abstract 2

Supplemental Digital Content is available in the text

## 1 Introduction

Gastric cancer (GC) is one of the most lethal malignancies, which poses a serious threat to the health of people worldwide,^[1,2]^ and it is the fifth most common malignancy.^[3–5]^ GC can significantly disrupt patients’ health status and reduce quality of life. Although the 5th highest number of new cancers all over the world, GC has the third highest rate of death.^[6]^ Most of all, in East Asia, the incidence and mortality of GC are the highest, especially in China, nearly 679,100 new GCs are diagnosed, and 498,000 patients die from GC annually.^[7]^ GC is a complex disease and its pathogenesis mechanism is not completely understood. And the current diagnostic system was proved to be relatively poor in early-stage diagnosis GC. Epidemiological studies have demonstrated that interaction of environmental elements and genetic elements has been found to contribute to the risk of GC. They have suggested that environmental exposures, such as unhealthy lifestyle, a salty diet, tobacco smoking, and *Helicobacter pylori* infection, have an effect on the development of GC.^[8]^ The most common cause is infection by the bacterium *H pylori*, which accounts for more than 60% of cases. Therefore, the fact that GC is a complex disease involving multifactorial etiology and gene-environment interactions has resulted in research efforts to identify individuals susceptible to GC. Single nucleotide polymorphisms (SNPs) are DNA sequence polymorphisms caused by variations in a single nucleotide at the genomic level. They are also the most frequent form of genetic variations in the human genome which can have an impact on cancer predisposition.^[9,10]^ Most SNPs are functionally neutral, but some have been found to alter gene expression and function, or be in linkage disequilibrium with causal loci associated with cancer risk and/or prognosis. The past decades have witnessed burgeoning research on SNPs associated with GC, and many gene SNPs derived from different approaches, such as inflammation, DNA repair, and microRNA-mediated silencing, have been described to affect individual susceptibility to GC. Most of these studies; however, have limited statistical power to detect small-effect SNPs and the results are often inconsistent and thus inconclusive. With the development of modern molecular biology, we have found the relationship between MIR155HG variants and GC susceptibility The expression of its locus and made certain achievements have provided the possibility for the diagnosis of GC.^[11]^ Building upon these studies, systematic reviews have evaluated the evidence regarding SNPs in individual genes or signaling pathways related to GC, but few reviews have comprehensively summarized and evaluated all SNPs related to GC. The objective of this study was to comprehensively evaluate significant SNPs associated with GC susceptibility. There is a lack of evidence to indicate which genetic model is most appropriate to identify associations of SNPs with GC; thus, instead of assuming the underlying genetic model, we applied various approaches to select the most appropriate genetic models of inherence and to measure the reliability of the associations.

## 2 Materials and methods

This study was conducted in accordance with the preferred reporting items for systematic reviews and meta-analyses guidelines and the protocol has been registering in the INPLASY database. Ethical approval will not be necessary since this systematic review and meta-analysis will not contain any private information of participants or violate their human rights.

### 2.1 Standards

This protocol will be performed to comply with the preferred reporting items for systematic reviews and meta-analyses protocols guidelines.

### 2.2 Ethical issues

Ethical approval was not necessary because this was a systematic review and meta-analysis based on published data.

### 2.3 Registration

The protocol has been registered on INPLASY.

### 2.4 Inclusion criteria

Case-control study, published in either English or Chinese that concern the susceptibility of the SNPs to the GC, will be integrated into this review. Studies published in full-text will be filtrated for containtion. All references that meet the criteria for inclusion are artificially selected to ensure that the relevant qualified documents are not omitted as much as possible. Studies were conducted worldwide, and Serum samples were taken from the study population before they received chemoradiotherapy.

### 2.5 Exclusion criteria

Literature types such as conference reports, review or review reports, and animal studies will be excluded. Studies that do not have enough data for genotype distribution calculations or that do not conform to the Hardy–Weinberg equilibrium (HWE) in the control group were excluded. Data extracted from the sample after chemoradiotherapy will not be included.

### 2.6 Participants

The GC samples included in the meta-analysis in this study were serum samples that had not received chemotherapy. The control group were healthy, nonmalignant diseases, and nonpancreatic cancer patients of any age (gender, country, tumor stage, etc).

### 2.7 Outcome

Gastric risk comparisons.

### 2.8 Search strategy

Studies published through January 2020 which be included in our meta-analysis were identified from PubMed, Web of Science, Embase, Cochrane Library, China National Knowledge Infrastructure, the Chinese Science and Technology Periodical Database, and Wanfang databases. There are no restrictions on literature language. The following search terms will be used for the search: “single nucleotide polymorphism,” “SNP,” “gastric cancer,” “stomach cancer.” Specific search terms and retrieval type about PubMed can be found in Supplementary Materials S1.

### 2.9 Data collection and analysis

**Figure 1:**
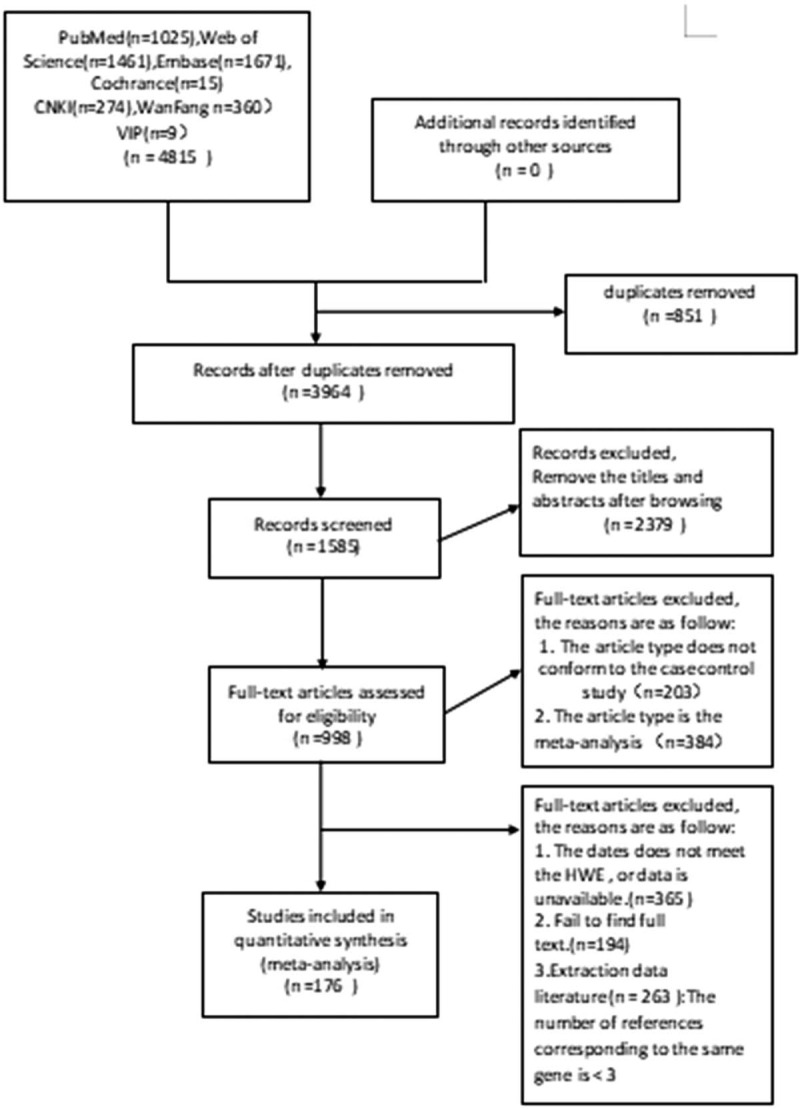
PRISMA flow diagram of literature search and selection. PRISMA = preferred reporting items for systematic reviews and meta-analyses.

The quality of the literature is first judged by 2 separate reviewers (ZY and QH) and, if disputed, by a third reviewer (JZ). The preferred reporting items for systematic reviews and meta-analyses flow diagram is shown in Figure 1. Data extracted from each paper included: author, country of publication, year, number of men and women, sample size, race, including genotype methods, genotype frequency, and HWE values. Yet for SNP that can be included in meta-analysis, the number of literature to be studied should be greater than or equal to 2. HWE is calculated using the goodness of fit test for each study's control group. For each meta-analysis, odds ratio (OR) with 95% confidence intervals will be calculated in fixed OR random effects. The fixed-effect model would be employed if *I*^2^ < 50%, which suggested low heterogeneity among include studies. Otherwise, random-effect model will be used. If we have enough great meaningful SNPs data, subgroup analysis would be used to analysis the source of the heterogeneity.

STATA software/MP 14.0 was applied to analyze the relationship between SNPs and GC. The random-effects model was used. The model parameters were estimated using the Markov chain Monte Carlo method of Gibbs sampling. The results are reported as the OR with 95% confidence intervals. To evaluate the inconsistency between direct and indirect effect estimates for the same comparisons, we evaluated each closed loop in the network. In a closed loop, we employed the inconsistency factor to evaluate heterogeneity among the included studies. To rank the SNPs and their gene models, we used the surface under the cumulative ranking probabilities. We further compared genetic models to select the most appropriate model using the algorithm by Thakkinstian et al.^[12]^ To assess the authenticity of the normally significant SNPs under the most appropriate genetic model determined by network meta-analysis or Thakkinstian’ algorithm, false positive report probability (FPRP) was calculated assuming 3 levels of prior probabilities (low: 0.1; moderate: 0.01; high: 0.001) and an OR of 1.5, as previously described.^[13,14]^ Significant SNPs with a FPRP value < 0.2 were considered noteworthy.^[14]^

Thakkinstian's algorithm was also used to evaluate the best model to be the SNP obtained from the previous optimal gene model in the reticular meta-analysis.^[12]^ We cannot judge the advantages and disadvantages of network meta-analysis and Thakkinstian’ algorithm according to the existing data, so we use FPRP to test the results of these 2 methods. According to study,^[13,14]^ in calculation of FPRP, the prior probabilities was be set as 3 levels. The low level of prior probabilities is 0.1, and for moderate and high level is 0.01 and 0.001. And we use moderate prior probabilities to calculate. FPRP value <0.2 were deemed for the significative SNPs.^[14]^

Diagnostic meta-analysis was conducted to determine sensitivity and specificity of SNPs in predicting GC risk using the Meta-DiSc software.^[15]^

### 2.10 Qualitative evaluations

The methodological quality of data was assessed based on the STrengthening the REporting of Genetic Association Studies statement.^[16]^ Two reviewers conducted the rating independently and a third reviewer was consulted for consensus if disagreement occurred.

### 2.11 Subgroup analysis

We will conduct a subgroup analysis of the SNPs most associated with GC, according to race, type of age, sex, and so on.

### 2.12 Sensitivity analysis

Sensitivity analysis will be conducted to check the robustness and reliability of pooled outcome results.

### 2.13 Assessment of publication biases

We will evaluate publication bias using the funnel plot as well as statistical tests (Egger test and Begg test).

## 3 Discussion

Risk association analysis based on a priori genetic model may be misleading if an inappropriate genetic model was assumed.^[17]^ In the study of correlation in GC risk, SNPs are effective methods to evaluate gene-gene and gene-environment interactions. By the end of our literature search in February 2020. We collected 1025 SNPs. This study did not make any assumptions, and observed the genotype significance of which gene models for GC susceptibility in a paired meta-analysis. To determine the most appropriate GC risk association model. Network meta-analysis and Thakkinstian algorithms were used. Those SNPs we obtained through analysis of our study may assist clinicians in assessing the prognosis of Gastric cancer (GC) patients and selecting appropriate targets therapy.^[18]^ It should be noted that the present study may have potential limitations of homogeneity as a result of the various race. And our meta-analysis may need additional large sample size, detailed PC risk factor data, and high-quality studies to explore the susceptibility between SNPs and the risk of GC.

## Author contributions

**Analysis planning:** Jing-Hui Zheng, Xiang-Dong Zhu, Chi Zhang.

**Conceptualization:** Jing-Hui Zheng, Xiang-Dong Zhu, Chi Zhang.

**Data curation:** Zhuo-Miao Ye, Qing-Yu Hu.

**Draft manuscript:** Zhuo-Miao Ye, Qing-Yu Hu.

**Investigation:** Jing-Hui Zheng.

**Manuscript editing:** Zhuo-Miao Ye, Jing-Hui Zheng.

**Methodology:** Zhuo-Miao Ye, Jing-Hui Zheng, Xiang-Dong Zhu.

## Supplementary Material

Supplemental Digital Content
