# Supplementary material for: A comprehensive evaluation of single nucleotide polymorphisms associated with gastric cancer risk: A protocol for systematic review and network meta-analysis
Source: Medicine (Baltimore). 2020 Jun 19;99(25):e20448. doi: 10.1097/MD.0000000000020448 (PMC7310972; doi:10.1097/MD.0000000000020448)
Supplement: Supplemental Digital Content [file medi-99-e20448-s001.docx]

Search **(((((((("Polymorphism, Single Nucleotide"[Mesh]) OR Nucleotide Polymorphism, Single[Title/Abstract]) OR Nucleotide Polymorphisms, Single[Title/Abstract]) OR Single Nucleotide Polymorphisms[Title/Abstract]) OR SNPs[Title/Abstract]) OR Single Nucleotide Polymorphism[Title/Abstract]) OR SNP[Title/Abstract])) AND (((((((((((((((((("Stomach Neoplasms"[Mesh]) OR Neoplasm, Stomach[Title/Abstract]) OR Neoplasms, Stomach[Title/Abstract]) OR Gastric Neoplasms[Title/Abstract]) OR Gastric Neoplasm[Title/Abstract]) OR Neoplasm, Gastric[Title/Abstract]) OR Neoplasms, Gastric[Title/Abstract]) OR Cancer of Stomach[Title/Abstract]) OR Stomach Cancers[Title/Abstract]) OR Gastric Cancer[Title/Abstract]) OR Cancer, Gastric[Title/Abstract]) OR Cancers, Gastric[Title/Abstract]) OR Gastric Cancers[Title/Abstract]) OR Stomach Cancer[Title/Abstract]) OR Cancer, Stomach[Title/Abstract]) OR Cancers, Stomach[Title/Abstract]) OR Cancer of the Stomach[Title/Abstract]) OR Gastric Cancer, Familial Diffuse[Title/Abstract])**
